# The effects of gelatin supplementation prior to cooling on ram semen quality and fertility

**DOI:** 10.21451/1984-3143-AR2017-0021

**Published:** 2018-08-16

**Authors:** Anderson Marques Pinto Bandeira, José Eduardo Matos, Alexandre Nízio Maria, Paulo César Falanghe Carneiro, Phillip H. Purdy, Hymerson Costa Azevedo

**Affiliations:** 1 Universidade Federal de Sergipe, São Cristóvão, .; 2 Embrapa Tabuleiros Costeiros, Empresa Brasileira de Pesquisa Agropecuária (EMBRAPA), , .; 3 Agricultural Research Service (ARS), , , .

**Keywords:** gelatinization, homogeneity, sperm sedimentation

## Abstract

The physical and chemical characteristics of gelatin have been used to justify its inclusion in extenders to preserve the sperm quality and improve results of cervical artificial insemination with cooled semen. The objective of this study was to evaluate the effects of gelatin supplementation in cooling extender on the quality and fertility of ram semen stored at 5°C. Semen samples (n = 24) of Santa Inês rams (n = 6) were diluted in Glycine-Yolk-Milk extender without (control) or with 1.5% of gelatin. The samples were loaded into 0.25 mL straws, cooled to 5°C and stored vertically for 48 and 72 hours. Sample quality was evaluated using straw homogeneity tests based on pH, osmolality and the proportion of spermatozoa (PS) in both upper and lower segments of straws (US and LS), analyses of sperm motility, plasma and acrosomal membrane integrity, and by fertility after artificial insemination. Differences between the US and LS of straws were found for pH and PS (%). They were significant only in the control group at both times: pH – 5.96 *vs.* 5.71 at 48 h and 6.13 *vs.* 5.89 at 72 h; PS – 21.66 *vs.* 78.34 at 48 h and 20.87 *vs.* 79.13 at 72 h. Storage in gelatin had very little, to no effect on the sperm kinetics or on the sperm membrane integrity evaluations. The addition of gelatin to the extender did not affect the pregnancy rate which ranged from 4.4 to 26.1%. We conclude that gelatin is effective in maintaining the physical and chemical homogeneity of the semen samples. Further research is needed in order to optimize the use of gelatin supplementation and elucidate any potential benefits.

## Introduction

Cooled semen has been used for many years as an important tool for the conservation, dissemination and transport of genetic sheep genetics ( Evans and Maxwell, 1990 ; Salamon and Maxwell, 2000 ). Recent research continues to use basic systems to cool semen to 4 to 15°C, diluents composed of whole, skim or reconstituted milk, Egg Yolk, Tris, Tes, fructose, glucose, citric acid and bovine serum albumin, or by commercial media such as OVIXcell (IMV technologies, France) which results in the ability to hold semen for 0 to 168 hours ( Allai *et al*., 2015 ; Quan *et al*., 2016 ; Benmoula *et al*., 2017 ; Hashem *et al*., 2017 ; Gheller *et al*., 2018 ; Falchi *et al*., 2018 ).

Cooling semen to 5°C can reduce the sperm metabolic activity and motility which will enable it to be stored prior to artificial inseminations – AI ( Evans and Maxwell, 1990 ). However, Nagy *et al*. (2002) , Rahman (2011) and El-Speiy *et al*. (2014) observed with rabbits and Salvador *et al*. (2006) observed with goats that the cooling and storage of liquid semen can promote sedimentation of sperm and diluent components leading to pH fluctuations and concentration of the metabolites. Consequently, the sperm quality when stored in an extender may depend on its position in the tube or straw, particularly when the samples are held in a vertical position prior to cooling or freezing.

Although most manuscript do not specify how the samples are positioned during cooling and transportation, many of the procedures keep the semen in a vertical position in glass or plastic tubes or even in vials such as those cited by Sousa and Bicudo (2003) and Yániz *et al*. (2005) . In addition, some automated semen refrigeration and freezing systems arrange the doses upright during processing ( Maia *et al*., 2009 ; Sicherle *et al*. 2011 ). All of these procedures and systems can lead to sedimentation of the sperm during sample processing, storage and cryopreservation.

It was suggested that supplementing semen extender with gelatin may prevent the sedimentation of sperm ( Nagy *et al*., 2002 ; Yániz *et al*., 2005 ) and preserve the homogeneity of the medium ( Santos *et al*., 2015 ). In addition, it was suggested that gelatin may protect boar sperm from cold shock ( Corcini *et al*., 2011 ), reduce the metabolic demand on ram sperm ( Yániz *et al*., 2005 ), increase the viscosity of cooled ram semen in such a way that it becomes temporarily solidified ( Yániz *et al*., 2005 ) which may then decrease the semen backflow from the cervix to the vagina during the AI ( Paulenz *et al*., 2010 ; Corcini *et al*., 2011 ).

Many of these hypotheses are assumed to be true but have not been fully tested. With sheep the glycine-yolk-milk cooling extender has been tested and shown to be efficient in preserving ram semen ( Gonzalez and Costa, 2012 ; Falleiros *et al*., 2013 ), but the addition of gelatin to this extender has never been tested. Often, supplementing extenders with components that were not part of the original recipe can result in interactions that may be deleterious. Consequently, the aim of the present study was evaluate the effect of gelatin supplementation in glycine-yolk-milk cooling extender on the quality and use of ram semen preserved at 5°C.

## Materials and Methods

All procedures have been carried out in accordance with the Conselho Nacional de Controle de Experimentação Animal (CONCEA), law 11794/2008 (CEUA - Embrapa Tabuleiros Costeiros - Protocol number: 18112015.0001) for use of experimental animals.

The experimental activities were conducted between May and November, in Sergipe State, Brazil, in tropical semi-arid region (Lat.10°36’09.4”South; Long.37°38’30.3”West).

### Semen collection and processing

Four ejaculates were collected from each of six Santa Inês rams using an artificial vagina, split and then diluted with either the control or gelatin-treated extender. A modified glycine-yolk-milk-medium - GYM ( Gonzalez and Costa, 2012 ) was used as the control and the treatment samples were diluted with the control medium supplemented with 1.5% commercial type B powder gelatin ( Yániz *et al*., 2005 ) produced from pre-treated bovine skin or ossein (Dr. Oetker™, Brazil).

The pH of the semen diluted with each extender was measured and the samples were then loaded into 0.25 ml straws containing 100 × 10^6^ spermatozoa. The straws were arranged in a vertical position and cooled from 32°C to 5°C at 0.22°C/min in a refrigerator designed to minimize an abrupt decrease in temperature due to contact of the straws with other surfaces. The cooling rate was measured using a digital thermometer BK Precision 710 (B&K Precision Corp., USA) connected to a temperature sensor (Type K thermocouple) inserted into a straw filled with extender lacking sperm to record the temperature every minute (Thermolink Software w/RS-232). The samples were maintained at 5°C for 48 and 72 hours and then assessed for *in vitro* and *in vivo* quality.

Only fresh semen samples with a minimum quality were used in this study: at least vigor score of 3.0 (0-5) and 80% motile spermatozoa (0-100%) both evaluated subjectively under optical microscopy (400X magnification) and, 80% normal spermatozoa, using the same technique used to evaluate the acrosome of sperm in the cooled semen. Some analyzes such as acrosome integrity and plasma membrane integrity were measured on fresh semen diluted with GYM in order to establish a baseline for these characteristics.

### Evaluation of semen homogeneity

Straws from both experimental groups were cut after cooling in order to divide their contents in upper (US) and lower (LS) segments. Contents of each segment were pooled by ram and transferred to microtubes for evaluation of pH (pH meter), osmolality (vapor pressure osmometer) and to determine the proportion of spermatozoa (PS) in each segment. Aliquots, 10μl, of the samples were diluted in 3.990µl of distilled water and the proportion of sperm in each segment was determined using a Neubauer chamber and a phase contrast microscope at 400X magnification. The semen homogeneity was determined by computing the differences between the US and LS for each treatment.

### Computerized assessment of sperm kinetics

The Sperm Class Analyzer (SCA™, Microptc, Barcelona, Spain) was used to evaluate the motility characteristics of the samples with the following system setup: temperature = 37°C; particle area between 30 to 70 μm^2^; VCL = 20 < slow < 100 < medium < 200 < rapid; progressivity > 80% of STR; circular < 50% LIN; VAP points = 5; connectivity = 12. Aliquots of cooled semen from the entire straw were diluted in evaluation solution (X-Cell™, IMV Technologies, France), to 16 x 10^6^ spermatozoa/ml. Aliquots (3 μl) of this solution were placed in a Makler chamber (Sefi-Medical Instruments, Haifa, Israel) and placed on the stage of a Nikon 50i microscope for image capture that included 500 to 600 cells/analysis (3 to 4 fields with 150 to 200 cells/field). The analyzed parameters were total motility (TM), progressive motility (PM), curvilinear velocity (VCL), straight-line velocity (VSL), average path velocity (VAP), linearity (LIN), straightness (STR), amplitude of lateral head displacement (ALH), and flagellar beat-cross frequency (BCF).

### Acrosome integrity

The percentage of spermatozoa with morphologically intact acrosomes (ACI) was determined following fixation of cells with 0.2% glutaraldehyde ( Blom, 1973 ). Samples (100 sperm/sample) were evaluated using phase contrast microscopy (Nikon 50i; 1000X magnification) to determine the percentage of spermatozoa without acrosomal wrinkling.

### Plasma membrane integrity

The technique described by Harrison and Vickers (1990) was used to evaluate the sperm plasma membrane integrity (PMI) using the combination of carboxyfluorescein diacetate (CFDA) and propidium iodide (PI). Aliquots of samples (25 μl) were diluted in 250 μl of evaluation solution (X-Cell™, IMV Technologies, France), and 10 μl of this mixture was transferred to a microtube containing staining solution (40 μl) composed of sodium citrate (3%), PI (7.3 μM) and CFDA (20.0 μM) in water. After incubation (10 min), 200 sperm per sample were counted under an epifluorescence microscope (Nikon Eclipse 50i; 1000X magnification; 505 nm emission filters). The spermatozoa were classified as intact plasma membrane - showing green fluorescent brightness (CFDA) over the head, including the acrosome area or with plasma membrane damaged - showing red fluorescent brightness (PI) on the head and /or the acrosome area.

### Artificial insemination

Santa Inês ewes (n =182) were assigned to four experimental groups (45 or 46 ewes/group) based on their age and body condition score (≥2.5 using the range of scores from 0 to 5; Russel *et al*., 1969 ). The estrous cycle of the ewes was synchronized using sponges (medroxyprogesterone acetate - MAP, Progespon™, Schering-Plough, Cotia, São Paulo, Brazil), cloprostenol sodium (Prostaglandin F2 alpha - PGF2α, Sincrocio™, Ouro Fino, Cravinhos, São Paulo, Brazil), and 400 IU of equine chorionic gonadotropin (eCG, Novormon™, Schering-Plough, Cotia, São Paulo, Brazil) according to the protocol described by Antunes-Melo *et al*. (2015) . The ewes were inseminated 50 hours after sponge removal and application of eCG, using a transcervical technique according to procedures described by Taqueda *et al*. (2011) . The ewes were inseminated with either the control (GYM without gelatin) or treated semen (GYM + 1.5% gelatin) that had been cooled for either 48 or 72 hours. Semen from each ram was used to inseminate either 30 ewes per ram (n = 4 rams) or 31 ewes per ram (n = 2 rams) for a total of 182 ewes.

The fertility was determined 39 to 48 days after the day of insemination using an ultrasound (Logic α100, General Electrics, Sao Paulo, Brazil) equipped with a 5 MHz transrectal transducer and the pregnancy rate (number of ewes pregnant per number inseminated per treatment group) was determined.

### Experimental design and statistical analysis

A randomized complete block design was used to analyze the *in vitro* data (n = 6 blocks or rams), with four replications within the blocks (n = 4 ejaculates/ram) totaling 24 replications. In the artificial insemination trial each experimental group had 45 or 46 replications represented by the number of ewes inseminated.

The statistical analyses were performed using the Shapiro-Wilk test to assess the normality of the distribution. Normal data (pH and PS at 72 h and PM, VSL, LIN, STR at 48 and 72 h) were analyzed using the analysis of variance General Linear Model (GLM), while the non-normal data (TM, VCL, VAP, ALH, BCF, ACI and PMI at 48 and 72 h, pH, Osmolality and SP at 48 h and Osmolality in 72 h) were analyzed using the Wilcoxon non-parametric test. Pearson’s Chi-Squared test was used to determine differences in pregnancy rates. A significance level of 0.05 was used and all analyses were performed using SPSS, version 15.0.

## Results

The evaluation of the semen dose homogeneity (pH, osmolality and proportion of spermatozoa by US and LS), is presented in Table 1 . The pH levels of the control (6.47) and gelatin-treated samples (6.35) decreased after being maintained at 5°C for 48 and 72 h in both segments of the semen straws. At both evaluation times differences (P < 0.05) between US and LS pH were observed in the control samples but not (P > 0.05) in the samples diluted in extender containing gelatin. The pH was lower (P < 0.05) in the LS than in the US in the control group. There were no differences (P > 0.05) between the segments for osmolality for both control and gelatin group at 48 or 72 h. The proportion of spermatozoa in the US and LS differed (P < 0.05) in the control but not (P > 0.05) in gelatin group regardless of the cooling time. There were higher (P < 0.05) sperm proportions in the lower segments in control group at 48 and 72 h.

**Table 1:** Semen dose homogeneity based on differences between upper (US) and lower (LS) segments of straws (mean ± standard deviation) with ram semen diluted in medium with 1.5% gelatin and cooled at 5°C for 48 and 72 hours.

Parameter	Segment	Cooling Time (hours)			
		48		72	
		Control	Gelatin	Control	Gelatin
pH	US	5.96 ± 0.33^a^	5.86 ± 0.28^a^	6.13 ± 0.20^a^	5.89 ± 0.21^a^
	LS	5.71 ± 0.31^b^	5.84 ± 0.32^a^	5.89 ± 0.25^b^	5.87 ± 0.21^a^
Osmolality (mOsm)	US	358.67 ± 10.13	367.25 ± 13.96	397.50 ± 47.29	435.13 ± 82.40
	LS	355.50 ± 8.97	371.92 ± 11.43	404.97 ± 31.41	410.63 ± 40.87
Sperm Proportion (%)	US	21.66 ± 16.18^b^	50.56 ± 4.79^a^	20.87 ± 18.92^b^	51.88 ± 8.76^a^
	LS	78.34 ± 16.18^a^	49.44 ± 4.79^a^	79.13 ± 18.92^a^	48.12 ± 8.76^a^

a,b Different letter indicates statistical difference (P < 0.05) between segments of the same experimental group and parameter

Storage in gelatin had very little or no effect on the sperm kinetic analysis ( Tab. 2 ) as well as on the sperm membrane integrity evaluations ( Tab. 3 ). While some statistical differences were observed, the meaning had no apparent biological significance.

**Table 2:** Sperm kinetics analysis (mean ± standard deviation) of ram semen diluted in medium with 1.5% gelatin and cooled at 5°C for 48 and 72 hours.

Kinetic Parameter	Group	Cooling Time (hours)	
		48	72
Total motility TM (TM, %)	Control	98.2 ± 2.7	96.6 ± 4.5^a^
	Gelatin	97.2 ± 3.0	92.9 ± 9.8^b^
Progressive motility (PM, %)	Control	58.0 ± 6.1^a^	49.6 ± 8.8^a^
	Gelatin	46.2 ± 4.0^b^	41.9 ± 8.7^b^
Curvilinear velocity (VCL, μm/s)	Control	357.0 ± 30.7^a^	325.3 ± 42.7
	Gelatin	336.4 ± 52.9^b^	321.1 ± 62.7
Straight-line velocity (VSL, μm/s)	Control	197.8 ± 20.6^a^	168.6 ± 25.0
	Gelatin	172.7 ± 18.0^b^	157.1 ± 26.2
Average path velocity (VAP, μm/s)	Control	249.5 ± 23.4^a^	221.6 ± 29.3
	Gelatin	229.3 ± 25.0^b^	214.0 ± 33.8
Linearity (LIN, %)	Control	55.5 ± 4.8	51.8 ± 3.6
	Gelatin	52.1 ± 6.4	49.7 ± 6.3
Straightness (STR, %)	Control	79.3 ± 3.5^a^	76.0 ± 4.1
	Gelatin	75.4 ± 2.8^b^	73.4 ± 3.5
Amp. of lateral head displ. (ALH, μm)	Control	3.0 ± 0.2	3.0 ± 0.2
	Gelatin	2.9 ± 0.4	2.9 ± 0.5
Flagellar beat-cross frequency (BCF, Hz)	Control	53.7 ± 2.0^a^	55.4 ± 2.3^a^
	Gelatin	51.1 ± 4.8^b^	52.7 ± 4.6^b^

a,b Means followed by different superscript letters indicate differences (P < 0.05) between groups within each cooling time.

**Table 3:** Sperm integrity evaluations (mean ± standard deviation) and fertility (mean) of ram semen diluted in medium with 1.5% gelatin and cooled at 5°C for 48 and 72 hours.

Parameter	Group	Cooling Time (hours)	
		48	72
Acrosome integrity (ACI, %)	Control	96.7 ± 2.9	96.6 ± 3.5
	Gelatin	96.2 ± 3.2	95.9 ± 3.4
Plasma membrane integrity (PMI, %)	Control	72.4 ± 13.9^b^	69.9 ± 15.0
	Gelatin	77.3 ± 13.4^a^	69.5 ± 16.4
Fertility (%)	Control	11.1	13.1
	Gelatin	26.1	4.4

a,b Means followed by different superscript letters indicate differences (P < 0.05) between groups within each cooling time.

After 48 hours, the gelatin-treated group exhibited lower values (P < 0.05) for PM, VCL, VSL, VAP, STR and BCF, and similar values (P > 0.05) for TM, LIN and ALH when compared to the control. After 72 hours, the gelatin–treated group had lower values (P < 0.05) of TM, PM and BCF, however, other parameters showed no differences (P > 0.05) between the groups at this cooling time: VCL, VAP, LIN, STR and ALH.

The fresh semen diluted with GYM presented PMI 87.43 ± 7.28% and ACI 99.35 ± 0.88% (mean and standard deviation). Gelatin did not affect (P > 0.05) the ACI of semen samples refrigerated for 48 hours but, compared to the control, the gelatin-treated group had higher (P < 0.05) PMI at this cooling time. No differences (P > 0.05) in acrosomal or plasma membrane integrity were observed in the semen cooled for 72 hours. Furthermore, pregnancy rates for semen cooled either with or without gelatin were similar (P > 0.05) at both times ( Tab. 3 ).

## Discussion

There are few reports on the use of gelatin with ram semen but it seems that the composition of the diluents, the proportion of gelatin, holding temperature, time of storage and state of the sperm sample (liquid or solid) may be responsible for the different effects of gelatin on the cooled semen quality and fertilizing potential. In this research, the gelatin reduced the sperm sedimentation and maintained the pH homogeneity of the cooled semen for 48 and 72 hours. The higher homogeneity may result in a more favorable environment because the evenly distributed buffers and sperm could reduce the magnitude of the natural occurring pH shifts ( Nagy *et al*., 2002 ; Salvador *et al*., 2006 ; Rahman, 2011 ; El-Speiy *et al*., 2014 ; Santos *et al*., 2015 ). Normally the semen pH level should be maintained between 6.0 and 6.5 to sustain the viability of ram sperm ( Bartoov *et al*., 1980 ). However, the natural production of lactic acid during the dilution and cooling phases of semen ( Dziekónska *et al*., 2009 ) reduce the levels of intra and extracellular pH ( Gadea, 2003 ; Yániz *et al*., 2011 ) which was also observed in this research. Furthermore, because of the greater homogeneity of the solution, the cryoprotectant systems may better protect the sperm against thermal shocks ( Holt, 2000 ; Corcini *et al*., 2011 ).

While the addition of gelatin to the extender was beneficial for maintaining a homogeneous environment, the obvious benefits for motion characteristics, membrane integrity and fertility were not apparent.

The addition of gelatin altered some of the sperm kinetic parameters in comparison to the control. Although the differences were significant, they do not seem to have much biological importance especially if we consider that sperm will be exposed to the uterine environment and may alter their movement. The sperm in the gelatinized media had more curvilinear or irregular movements, evidenced by lower values of VSL, VAP and STR after 48 hours of storage. These motility patterns were similar to rabbit sperm diluted with gelatinized extender and refrigerated for 0 to 96 hours ( López-Gatius *et al*., 2005 ). These observations are also consistent with Mortimer (1997) who reported that, the higher the viscosity of diluent, the lower the flagellar movement amplitude. Hirai *et al*. (1997) also observed that the proportion of progressively motile bull sperm, and their velocity, was reduced in highly viscous media. Perhaps, if analyses of these characteristics were performed immediately after collection and dilution and after 24 hours of holding at 5°C, then an apparent benefit may have been observed.

We speculate that the reported variation in quality and fertility following gelatin supplementation may be attributed to a modulation of sperm capacitation by gelatin. During capacitation, binder of sperm proteins (BSP) from the seminal plasma interact with plasma membrane proteins and induce or regulate the capacitation process ( Arangasamy and Singh, 2007 ; Arangasamy, 2010 ; Plante *et al*., 2016 ). Furthermore, all known BSP proteins will interact with gelatin but this does not have an apparent biological significance ( Plante *et al*., 2016 ). Consequently, if a semen sample is supplemented with gelatin then the addition of this protein may occupy the BSPs thus altering or inhibiting capacitation. Following the same line, the gelatin can hypothetically play an important influence on the BSPs roles in sperm motility and viability, in the formation of the oviductal sperm reservoir, in the regulation of cell volume and possibly in the interaction between sperm and oocytes ( Plante *et al*., 2016 ).

Therefore, we conclude that gelatin is effective in maintaining the physical and chemical homogeneity of a semen sample when held for prolonged periods of time at 5°C but the effects of gelatin supplementation on cooled ram sperm used for artificial insemination are still to be determined, especially considering that a small sample size of ewes were utilized in this study. Optimization of this treatment and further studies to confirm our results with higher number of samples are obviously needed to elucidate the gelatin effects on sperm physiology and fertility.

## References

[B001] Allai L, Druart X, Contell J, Louanjli N, Moulaa AB, Badi A, Essamadi A, Nasser B, El Amiri B (2015). Effect of argan oil on liquid storage of ram semen in Tris or skim milk based extenders. *Anim Reprod Sci*.

[B002] Antunes-Melo KD, Oliveira VS, Santos ADF, Oliveira CA, Mendonça LM, Goveia JSS, Almeida TS (2015). Use of reduced doses of eCG applied by different routes in the TAI program in Santa Ines sheep. *Semin Ciências Agrárias*.

[B003] Arangasamy A, Singh LP (2007). In-vitro capacitation of epididymal spermatozoa with heparin and gelatin binding buffalo
seminal plasma proteins. *Indian J Anim Sci*.

[B004] Arangasamy A. (2010). Effect of stallion seminal plasma proteins on in-vitro capacitation of equine spermatozoa. *Indian J Anim Sci*.

[B005] Bartoov B, Bar-Sagie D, Mayevsky A. (1980). The effect of pH on ram sperm collective motility driven by mitochondrial respiration. *Int J Androl*.

[B006] Benmoula A, Badi A, El Fadili M, El Khalil K, Allai L, El Hilali A, El Amiri B. (2017). Effect of season on scrotal circumference, semen characteristics, seminal plasma composition
and spermatozoa motility during liquid storage in INRA180 rams. *Anim Reprod Sci*.

[B007] Blom E. (1973). The ultrastructure of some characteristic sperm defects and a proposal for a new classification
of the bull spermiogram. *Nord Vet Med*.

[B008] Corcini CD, Moreira F, Pigozzo R, Varela AS, Torres NU, Lucia T (2011). Semen quality and reproductive performance after artificial insemination with boar sperm
stored in a gelatin-supplemented extender. *Livest Sci*.

[B009] Dziekońska A, Fraser L, Strzeżek J. (2009). Effect of different storage temperatures on the metabolic activity of spermatozoa following
liquid storage of boar semen. *J Anim Feed Sci*.

[B010] El-Speiy ME, Elkomy AE, Kamel KI (2014). Effect of adding protein high viscosity (gelatin) in Tris extender on semen conservation
status, fertility rates, antioxidant status and sex ratio of rabbits. *Glob Vet*.

[B011] Evans G, Maxwell WMC (1990). Inseminación artificial de ovejas y cabras.

[B012] Falchi L, Galleri G, Zedda MT, Paua S, Boglioloa L, Ariua F, Ledda S (2018). Liquid storage of ram semen for 96 h: effects on kinematic parameters, membranes and DNA
integrity, and ROS production. *Livestock Science*.

[B013] Falleiros MB, Bicudo SD, Rodello L, Monteiro CD, Sakashita SM, Weiss RR (2013). Implicações do uso do extrato de LBD (lipoproteínas de baixa densidade)
ou da gema de ovo “purificada”, sobre a motilidade e morfologia espermáticas
no sêmen ovino refrigerado por 24 ou 48 horas. *Vet Zootec*.

[B014] Gadea J. (2003). Review: Semen extenders used in the artificial insemination of swine. *Span J Agric Res*.

[B015] Gheller SMM, Corcini CD, Jorgea P, Guilherme R, Thomaz LJ, Fabiana M, Varela AS (2018). Gelatin protects ram semen stored for 72 h at 5°C. *Small Rumin Res*.

[B016] Gonzalez CIM, Costa JAA (2012). Reprodução assistida e manejo de ovinos de corte.

[B017] Harrison RAP, Vickers SE (1990). Use of fluorescent probes to assess membrane integrity in mammalian spermatozoa. *J Reprod Fertil*.

[B018] Hashem EZ, Haddad R, Eslami M (2017). Evaluation of ram semen enrichment with oleic acid on different spermatozoa parameters
during low temperature liquid storage. *Small Rumin Res*.

[B019] Hirai M, Cerbito WA, Wijayagunawardane MPB, Braun J, Leidl W, Ohosaki K, Matsuzawa T, Miyazawa K, Sato K (1997). The effect of viscosity of semen diluents on motility of bull spermatozoa. *Theriogenology*.

[B020] Holt WV (2000). Basic aspects of frozen storage of semen. *Anim Reprod Sci*.

[B021] López-Gatius F, Sances G, Sancho M, Yániz J, Santolaria P, Gutiérrez R, Núñez M, Núñez J, Soler C. (2005). Effect of solid storage at 15°C on subsequent motility and fertility of rabbit semen. *Theriogenology*.

[B022] Maia MS, Bicudo SD, Azevedo HC, Sicherle CC, Sousa DB, Rodello L (2009). Motility and viability of ram sperm cryopreserved in a Tris-egg yolk extender supplemented
with anti-oxidants. *Small Rumin Res*.

[B023] Mortimer ST (1997). A critical review of the physiological importance and analysis of sperm movement in mammals. *Hum Reprod Update*.

[B024] Nagy S, Sinkovics G, Kovács A. (2002). Viability and acrosome integrity of rabbit spermatozoa processed in a gelatin-supplemented
extender. *Anim Reprod Sci*.

[B025] Paulenz H, Ǻdnøy T, Fossen OH, Söderquist L (2010). Effect on field fertility of addition of gelatine, different dilution rates and storage
times of cooled ram semen after vaginal insemination. *Reprod Dom Anim*.

[B026] Plante G, Prud’homme B, Fan J, Lafleur M, Manjunath P. (2016). Evolution and function of mammalian binder of sperm proteins. *Cell Tissue Res*.

[B027] Quan GB, Wu GQ, Wang YJ, Li DJ, Ma Y, Hong QH (2016). Effects of the Tris, Tes, or skim milk based extender on *in vitro* parameters
of ram spermatozoa during liquid storage. *Small Rumin Res*.

[B028] Rahman SAHA (2011). Effect of gelatin addition to extender on semen quality of rabbit. *Al- Mustansiriya
*. *J Sci*.

[B029] Russel AJF, Doney JM, Gunn RG (1969). Subjective assessment of body fat in sheep. *J Agric Sci*.

[B030] Salamon S, Maxwell WMC (2000). Storage of ram semen. *Anim Reprod Sci*.

[B031] Salvador I, Yániz J, Viudes de Castro MP, Gómez EA, Silvestre MA (2006). Effect of solid storage on caprine semen conservation at 5ºC. *Theriogenology*.

[B032] Santos FCC, Corcini CD, Costa VGG, Gheller SMM, Nogueira CEW, da Rosa Curcio B, Varela Jr AS. (2015). Effect of solid medium during cooled storage on stallion sperm parameters. Cryo Letters.

[B033] Sicherle CC, Maia MS, Bicudo SD, Rodello L, Azevedo HC (2011). Lipid peroxidation and generation of hydrogen peroxide in frozen-thawed ram semen supplemented
with catalase or Trolox. *Small Rumin Res*.

[B034] Sousa DB, Bicudo SD (2003). Inseminação artificial com sêmen ovino refrigerado por 24 horas
e transportado no sistema Equitainer. *Rev Bras Reprod Anim*.

[B035] Taqueda GS, Azevedo HC, Santos EM, Matos JE, Bittencourt RF, Bicudo SD (2011). Influence of anatomical and technical aspects on fertility rate based on sheep transcervical
artificial insemination performance. *Ars Vet*.

[B036] Yániz J, Martí JI, Silvestre MA, Folch J, Santolaria P, Alabart JL, López-Gatius F (2005). Effects of solid storage of sheep spermatozoa at 15ºC on their survival and penetrating
capacity. *Theriogenology*.

[B037] Yániz JL, Mateos JA, Santolaria P (2011). Zwitterionic buffers preserve ram semen quality more efficiently than TRIS during storage
at 15°C. *Small Rumin Res*.

